# Von Hippel-Lindau syndrome presenting as a lateral ventricular space-occupying lesion: a case report

**DOI:** 10.3389/fonc.2026.1899311

**Published:** 2026-09-15

**Authors:** Lu Liu, Yifei Chen, Zhaohui Zhang, Gaosheng Zhou

**Affiliations:** 1 The First College of Clinical Medical Science, China Three Gorges University, Yichang, China; 2 Department of Critical Care Medicine, Yichang Central People’s Hospital, Yichang, China; 3 Critical Care Medicine Laboratory, Yichang Sepsis Clinical Research Center, Yichang, China; 4 Laboratory of Critical Care Medicine, Zhongnan Hospital, Hubei Provincial Clinical Research Center for Critical Care Medicine (Sepsis Research Collaborative Unit), Yichang, China; 5 Department of Medical Administration, Yichang Central People’s Hospital, Yichang, China

**Keywords:** central nervous system neoplasms, hemangioblastoma, lateral ventricle, metastasis, VHL syndrome

## Abstract

Von Hippel-Lindau disease is a rare autosomal dominant genetic disorder caused by mutations in the *VHL* tumor suppressor gene located on chromosome 3. Its core feature is the development of vascular-rich tumors or cysts in multiple organ systems at different ages, mainly manifesting as cerebellar hemangioblastoma, retinal hemangioblastoma, renal cell carcinoma and/or multiple renal cysts, and adrenal pheochromocytoma, among others. The disease is rare and involves multiple systems, making diagnosis challenging. Genetic testing has gradually become the gold standard for its diagnosis. This case report describes a 54-year-old male patient who presented with an intracranial space-occupying lesion detected during a health check-up three years earlier, as well as headache, dizziness, and decreased vision for more than four months. Imaging and pathological examinations ultimately confirmed a diagnosis of VHL syndrome, complicated by a rare “collision tumor” composed of an intracranial (lateral ventricular) hemangioblastoma and metastatic clear cell renal cell carcinoma. After resection of the lateral ventricular tumor, the patient developed extensive intracranial hemorrhage and fatal brainstem hemorrhage due to the inherently high vascularity of the hemangioblastoma, and eventually died. Through this complex and high-risk case, we discuss the diagnostic difficulties of VHL syndrome, the surgical risks of central nervous system lesions, and multidisciplinary management strategies, aiming to provide practical guidance for clinical practice.

## Introduction

Von Hippel-Lindau disease (VHL) is a rare autosomal dominant disorder caused by germline deletion or mutation of the *VHL* gene, with a prevalence of approximately 1/47,000. It is characterized by multicentricity, multiplicity of tumors, and familial inheritance (1). The disease is driven by germline mutations in the *VHL* tumor suppressor gene located on chromosome 3p25-26, leading to loss of function of the VHL protein. This results in abnormal accumulation of downstream hypoxia-inducible factor (HIF), promoting angiogenesis and tumorigenesis (2). The most common manifestations of VHL, which can present across all age groups, are hemangioblastomas (HBs) of the central nervous system and spinal cord, followed by renal cell carcinoma, pancreatic tumors, pheochromocytoma, endolymphatic sac tumors, and cystadenomas of the broad ligament or epididymis (3). Within the central nervous system, HBs are predominantly located in the cerebellum (50–60%), spinal cord (40–50%), and brainstem (10–20%), while involvement of the pituitary stalk is rare (4). Symptoms vary according to tumor location and may include cerebellar signs (ataxia, cognitive impairment), cranial nerve dysfunction, spinal cord compression, or gradual vision loss due to retinal involvement (5). In recent years, with a deeper understanding of the VHL/HIF pathway, targeted agents such as the hypoxia-inducible factor-2α (HIF-2α) belzutifan has been approved by the FDA for adult patients with VHL disease who require therapy for associated renal cell carcinoma, central nervous system hemangioblastomas, or pancreatic neuroendocrine tumors not requiring immediate surgery. The phase 2 LITESPARK-004 trial demonstrated meaningful antitumour activity across all three tumor types, including CNS hemangioblastomas, bringing a revolutionary change in disease management (6). Nevertheless, surgical resection remains the primary treatment for established, symptomatic, or rapidly progressive central nervous system lesions (7). Here we report a complex case of a 54-year-old adult patient initially presenting with an intracranial mass that was ultimately diagnosed as VHL syndrome and resulted in death due to severe postoperative complications. We also discuss the comprehensive management of the disease in the context of recent literature.

## Case presentation

A 54-year-old male patient with no significant past medical history of hypertension, diabetes, or other comorbidities was found to have an intracranial space-occupying lesion during a routine health check-up three years ago. He had a long-standing history of smoking and alcohol consumption for over 30 years, with no documented family history of central nervous system tumors or other VHL-related pathologies. During that period, he experienced no significant dizziness, headache, nausea, vomiting, or facial pain and numbness, nor any other notable discomfort. His baseline performance status was consistent with an Eastern Cooperative Oncology Group (ECOG) performance status (PS) of 0-1, as he was fully ambulatory and capable of all self-care activities. Therefore, the finding was not specifically investigated or treated. In April 2024, the patient began to develop intermittent headache, dizziness, and progressive bilateral vision loss. For diagnostic clarification, contrast-enhanced whole-body CT and cranial MRI were performed in August 2024 on an outpatient basis; no steroid therapy was administered, and GCS/KPS scores were not formally documented at that time. The CT revealed left renal absence (consistent with prior nephrectomy), right renal multiple simple cysts without evidence of active malignancy, a left adrenal nodule (2.1 cm, thin peripheral enhancement), pancreatic atrophy with multiple cystic lesions and two early-enhancing nodules in the pancreatic head (suspected neuroendocrine tumors), and hepatic cysts—findings suggestive of VHL syndrome. The MRI demonstrated a left lateral ventricular nodule (1.8×1.9×2.1 cm) with marked enhancement and new small enhancing nodules in the left basal ganglia, left cerebellar hemisphere, vermis, and retro-medullary region. The differential diagnosis at this stage included hemangioblastoma, metastatic lesions, meningioma, and glioma, with VHL-related lesions considered most likely given the multisystem involvement (**Figure 1**). Further medical history taking revealed that the patient had undergone surgery for “renal cancer” in 2017 (specific pathology unknown). However, as this information was obtained solely from the patient’s and family’s oral history, no medical records from the outside institution could be retrieved; therefore, the specific stage at diagnosis, adjuvant treatments, and follow-up status for RCC could not be ascertained. CT imaging at presentation confirmed left renal absence consistent with prior nephrectomy; the right kidney showed multiple simple cysts with no enhancing solid components, and no evidence of active malignancy, local recurrence, or metastases was observed. The patient and family did not report any family history of VHL or related tumors, and germline VHL gene testing was not performed during the patient’s lifetime. In light of these multisystem tumor manifestations, a clinical diagnosis of VHL syndrome was made on clinical grounds.

**Figure 1:**
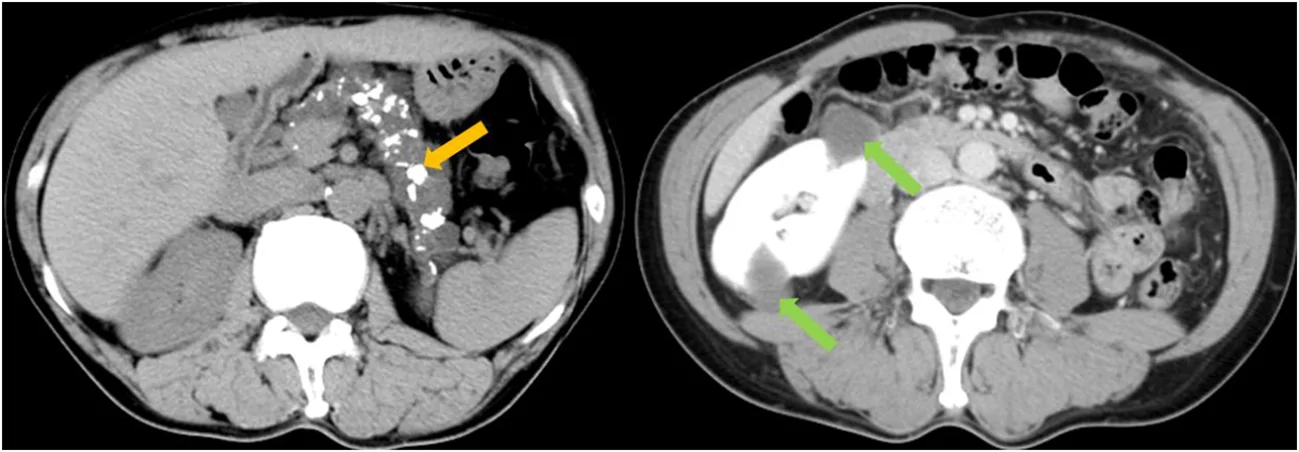
Two abdominal CT scan slices show the pancreas and right kidney. The left image shows multiple cystic hypodense lesions in the pancreas (orange arrows). The right image shows multiple simple cysts in the right kidney (yellow arrows).

Upon admission in October 2024, the patient’s GCS was 15 (E4V5M6) and KPS was 80. Preoperative laboratory tests revealed: white blood cell count 19.76×10^9^/L, hemoglobin 128 g/L, platelets 127×10^9^/L; PT 14.3s, APTT 36.1s, Fib 2.39 g/L, INR 1.09; creatinine 87 μmol/L, ALT 50 U/L, AST 43 U/L, CRP 29.97 mg/L. These findings were generally acceptable for surgery. No multidisciplinary discussion with renal or UGI oncologists was undertaken before surgery—a limitation we acknowledge. Because follow-up imaging performed on admission in October 2024 revealed rapid growth of the left lateral ventricular lesion (**Figure 2**), and in order to relieve the mass effect and prevent further neurological deterioration, the patient underwent “resection of the lateral ventricular space-occupying lesion plus external ventricular drainage” in late October 2024 after thorough communication with his family. The surgery proceeded without intraoperative complications, and gross total resection of the tumor was documented intraoperatively. However, no confirmatory postoperative imaging was obtained before the patient’s deterioration on the first postoperative day, when he developed fever (peak temperature 39 °C) along with an altered level of consciousness, progressing from lethargy to shallow coma. Emergency head CT revealed significantly increased hemorrhage in the surgical area and ventricular system compared with previous images, as well as a new hemorrhage involving the brainstem (**Figure 3**). Although emergency lumbar puncture was performed to release bloody cerebrospinal fluid and the drainage tube was adjusted, the patient’s condition continued to deteriorate, with signs of brain herniation. That night, the patient’s consciousness deepened to coma, and an urgent “bilateral external ventricular drainage” procedure was performed. After the second surgery, the patient was transferred to the intensive care unit in a state of deep coma, with a Glasgow Coma Scale score of E1V1M1, bilateral fixed and dilated pupils with absent light reflex, and severe brainstem failure requiring high-dose vasopressors to maintain blood pressure and mechanical ventilation for respiratory support. Despite full supportive treatment, the prognosis was extremely poor. The family decided to withdraw active treatment on the second day after surgery, and the patient subsequently died.

**Figure 2:**
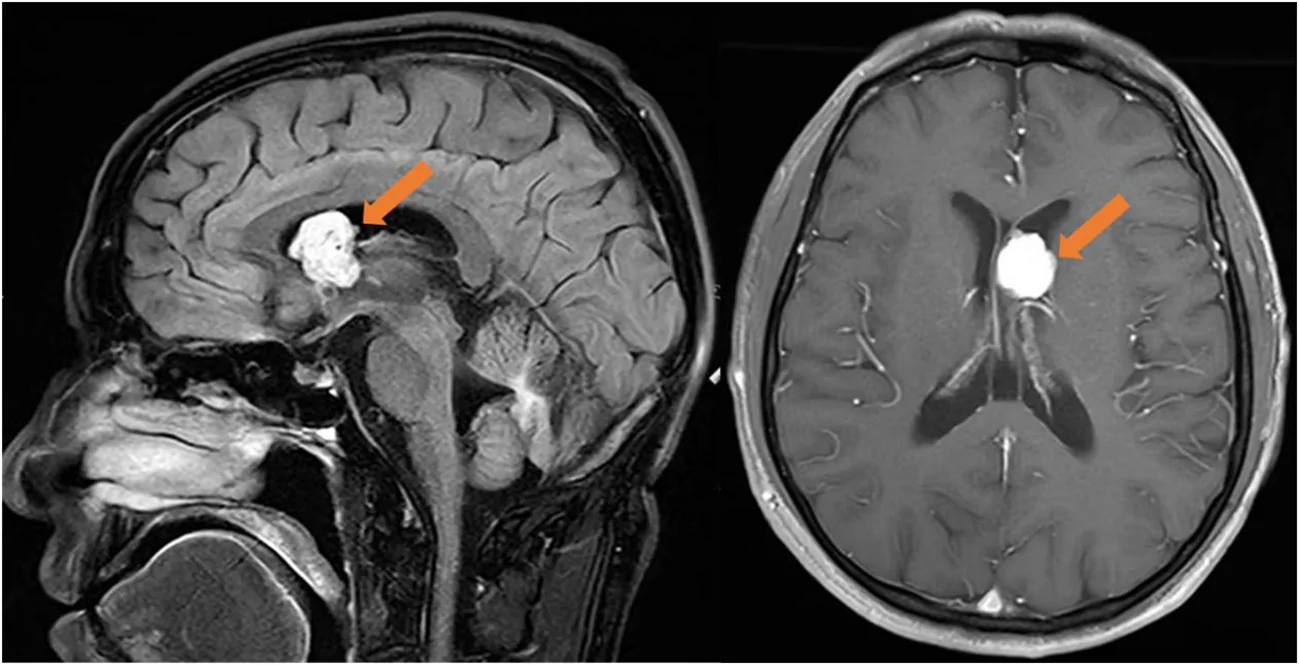
Axial T1-weighted contrast-enhanced MRI showing an irregularly shaped enhancing nodule in the left lateral ventricle (orange arrow).

**Figure 3:**
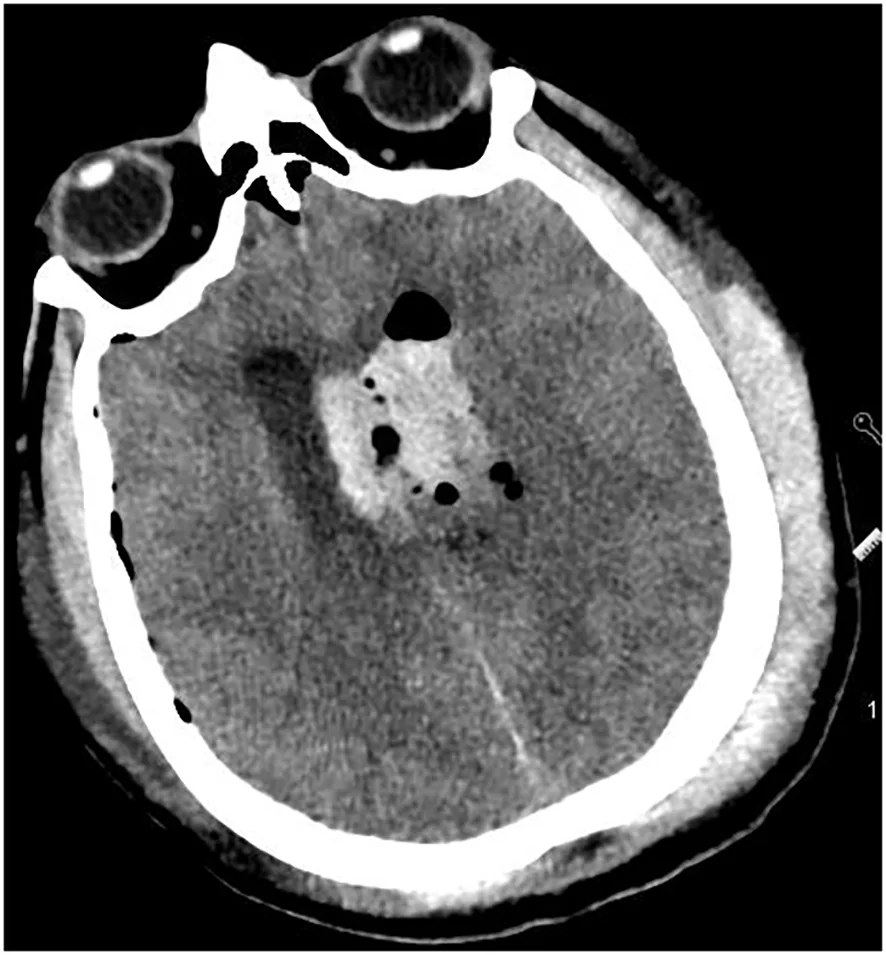
Non-contrast axial head CT showing hemorrhage in the surgical area and ventricular system, pneumocephalus, and a new hemorrhage involving the brainstem.

Microscopic examination of the submitted intracranial tumor tissue showed an admixture of two morphologically and immunophenotypically distinct tumor components. One component consisted of stromal cell clusters with abundant capillary networks, which were immunopositive for inhibin-A and neuron-specific enolase, consistent with a typical hemangioblastoma. The other component was composed of clear cells arranged in nested or acinar patterns, which showed positive immunostaining for Paired box protein 8, Carbonic anhydrase IX, and Cluster of differentiation 10, consistent with clear cell renal cell carcinoma. The two tumor components were closely juxtaposed, forming a so-called collision tumor. This finding confirmed that the intracranial mass was not a solitary VHL-associated hemangioblastoma, but rather a mixed tumor comprising a brain metastasis from renal cell carcinoma.

## Discussion

VHL syndrome is an autosomal dominant cancer predisposition disorder caused by mutations in the *VHL* tumor suppressor gene located on the short arm of chromosome 3 (3p25-26) (8). It is characterized by benign and malignant tumors in various organ systems as well as multiple organ cysts (9). Approximately 60–80% of patients with VHL syndrome develop central nervous system HBs. We acknowledge that germline VHL gene sequencing was not performed in this patient, and therefore the diagnosis was established on clinical grounds rather than molecular confirmation. This represents a limitation of our case report. Nevertheless, the patient met the established clinical diagnostic criteria for VHL syndrome, including a history of renal cell carcinoma, a central nervous system hemangioblastoma, and multiple visceral lesions (pancreatic cysts, renal cysts, and adrenal mass). While genetic testing remains the gold standard, the clinical phenotype in this case was highly characteristic and sufficient to support the diagnosis (**Table 1**). The pathological finding of an intracranial “collision tumor” is particularly rare (10). Only sporadic case reports have described clear cell renal cell carcinoma metastasizing into a central nervous system HB in a VHL patient (6, **Table 2**). The mechanism underlying this “tumor-within-a-tumor” phenomenon remains incompletely understood; it may be related to the exceptionally abundant blood supply of such HBs, which provides a “soil” for circulating tumor cells (11). This finding serves as a reminder that when VHL patients develop new or rapidly enlarging central nervous system lesions, the possibility of metastasis should be considered in the differential diagnosis, especially in those with a history of renal cell carcinoma.

**Table 1:** Clinical diagnostic criteria for VHL syndrome vs. present case.

Diagnostic criterion	Standard VHL criteria	Findings in the present case
Family history	Positive family history of VHL (1st- or 2nd-degree relative)	No family history of VHL or related tumors
For patients with a family history	≥1 VHL-related manifestation (CNS HB, retinal HB, RCC, or pheochromocytoma)	N/A (no family history)
For patients without a family history	≥2 VHL-related manifestations, one of which should be a hemangioblastoma	CNS hemangioblastoma (lateral ventricular) + history of renal cell carcinoma + multiple visceral lesions (pancreatic cysts, renal cysts, adrenal mass)
VHL-related manifestations recognized	Retinal HB, CNS HB, RCC, pheochromocytoma, PNET, ELST, multiple kidney cysts, multiple pancreatic cysts	CNS HB (✓), RCC (✓ by history), multiple kidney cysts (✓), multiple pancreatic cysts (✓), adrenal lesion (possible pheochromocytoma or adenoma)
Genetic testing	Germline *VHL* gene mutation (gold standard)	Not performed (clinical diagnosis only)
Clinical diagnosis conclusion	Meets criteria if ≥2 manifestations present without family history	Meets clinical criteria: CNS HB + RCC history + multiple visceral cysts → clinical diagnosis of VHL syndrome established

CNS, central nervous system; ELST, endolymphatic sac tumor; PNET, pancreatic neuroendocrine tumor; RCC, renal cell carcinoma.

**Table 2:** Summary of published cases of RCC metastasizing to CNS hemangioblastoma in VHL patients.

No.	Title	Year	Authors	DOI
1	Metastasis of renal cell carcinoma to central nervous system hemangioblastoma in two patients with von Hippel-Lindau disease	2001	Hamazaki S, et al.	10.1046/j.1440-1827.2001.01298.x
2	Spinal hemangioblastoma containing metastatic renal cell carcinoma in von Hippel-Lindau disease: case report and review of the literature	2005	Altinoz MA, et al.	10.3171/spi.2005.3.6.0495
3	Renal cell carcinoma to haemangioblastoma metastasis: a rare manifestation of Von Hippel-Lindau syndrome	2015	Dessauvagie BF, et al.	10.1016/j.jocn.2014.04.024
4	Metastasis of Renal Cell Carcinoma to Spinal Hemangioblastoma in a Patient with von Hippel–Lindau Disease: A Case Report	2021	Horiguchi K, et al.	10.3171/2021.5.FOCUS21308
5	Supratentorial Collision Tumor of Hemangioblastoma and Metastatic Clear Cell Renal Cell Carcinoma in a Patient with von Hippel-Lindau Disease	2023	Luo W, Sun C, Yu S	10.1159/000531876
6	A Cerebellar Tumor-to-Tumor Metastasis in a Patient With Von Hippel-Lindau Disease	2024	Wang T, et al.	10.1097/PAI.0000000000001197
7	The gradual expansion of multiple intramedullary metastatic renal cell carcinoma in a patient with von Hippel–Lindau disease with multiple intramedullary hemangioblastomas: illustrative case	2025	Matsuda M, et al.	10.3171/2025.2.FOCUS2512

The biological behavior of central nervous system HBs is unpredictable, alternating between stable phases and phases of rapid tumor or cyst growth (12). Surgical resection is the gold standard for treating symptomatic or progressive HBs. However, VHL-associated HBs are characterized by extremely rich vascularity, and intraoperative or postoperative bleeding is a major risk (13). A 2023 nationwide retrospective study from Japan involving 676 patients who underwent HB surgery showed that while perioperative functional deterioration rates and mortality did not differ significantly between VHL-associated and sporadic HBs, the surgical risks were substantial (14). The devastating intracranial and brainstem hemorrhage that occurred postoperatively in our patient directly led to his death. We believe this catastrophic bleeding was likely multifactorial, resulting from the inherently high vascularity of the hemangioblastoma, the additional vascular fragility contributed by the coexisting metastatic clear cell RCC component, and the surgical manipulation of this highly vascular collision tumor—both HB and RCC being recognized as bleeding-prone neoplasms. This case confirms the compounded hemorrhagic risk when two highly vascular tumor types coexist in the same intracranial lesion and highlights the importance of conducting a thorough preoperative vascular assessment and fully discussing the benefits and risks of surgery with the patient and the family. It should be emphasized that our patient was symptomatic with progressive neurological decline and significant mass effect, which justified surgical intervention—this case does not pertain to prophylactic surgery in asymptomatic individuals. For truly asymptomatic small tumors located in critical functional areas such as the brainstem or spinal cord, the current consensus favors regular, close follow-up rather than prophylactic surgery (15).

In recent years, the HIF-2α inhibitor Belzutifan has changed the treatment landscape for VHL syndrome (16). In 2021, the U.S. Food and Drug Administration approved Belzutifan for adult patients with VHL syndrome-associated renal cell carcinoma, central nervous system HBs, or pancreatic neuroendocrine tumors (17). Based on long-term follow-up data from clinical trials, Belzutifan has demonstrated objective response rates of 70%, 50%, and 90% for VHL-associated renal cell carcinoma, central nervous system HBs, and pancreatic neuroendocrine tumors, respectively, and significantly reduces the need for surgical intervention. Unlike tyrosine kinase inhibitors and immune checkpoint inhibitors—which are primarily used for advanced/metastatic RCC with limited evidence in VHL-associated HBs—belzutifan offers targeted HIF-2α inhibition with oral administration and a favorable safety profile. However, for symptomatic lesions with mass effect or hemorrhage risk, surgery remains the preferred option due to the need for rapid decompression (18). A hypothetical consideration is whether preoperative belzutifan could have altered the outcome in our patient. By reducing tumor vascularity and size, belzutifan might theoretically facilitate safer surgical resection in highly vascular lesions (19). However, this remains speculative, as no prospective data currently support neoadjuvant belzutifan for central nervous system hemangioblastomas. In our case, the rapid symptom progression and significant mass effect in the lateral ventricle, with impending risk of obstructive hydrocephalus, precluded any delay in surgical intervention. Therefore, preoperative systemic therapy was not feasible in this particular case. The decision between upfront surgery and systemic therapy should be individualized, taking into account symptom severity, tumor location, growth kinetics, patient performance status, and patient preference. For asymptomatic or slowly progressive lesions, stereotactic radiosurgery (SRS) may also offer a reasonable alternative to defer surgery. SRS is particularly valuable for lesions in surgically inaccessible locations or in patients with multiple lesions; recent meta-analyses have demonstrated pooled local tumor control rates of approximately 89% for CNS hemangioblastomas treated with SRS, with even higher rates observed in VHL-associated lesions (20). However, for symptomatic lesions with mass effect or risk of hemorrhage, surgery remains the preferred option. In our patient, the progressive symptoms and significant mass effect with impending obstructive hydrocephalus precluded SRS as a primary approach, as it does not provide immediate decompression. However, for patients who decline surgery despite progressive symptoms, systemic treatment or SRS may be considered if clinically appropriate (21).

## Conclusion

VHL syndrome is a complex hereditary tumor syndrome requiring lifelong management. This case of postoperative death due to bleeding in a patient with an intracranial collision tumor (hemangioblastoma combined with renal cell carcinoma metastasis) profoundly illustrates the diagnostic and therapeutic dilemmas faced in the advanced stages of this disease, especially regarding its central nervous system manifestations. It serves as a reminder to clinicians that in patients with multisystem tumors, a high index of suspicion for VHL syndrome should always be maintained, and early genetic confirmation should be pursued to guide family screening. When managing VHL-associated hemangioblastomas, the bleeding risk differs across management strategies. Theoretically, a collision tumor comprising both HB and RCC within the same lesion may further increase bleeding risk, as both tumor types are highly vascular—RCC being recognized as a bleeding-prone neoplasm—and their coexistence could compound intrinsic vascular fragility. This hypothesis, however, lacks direct evidence and warrants further investigation. Surgery carries the highest immediate perioperative risk due to extreme tumor vascularity, whereas active surveillance is associated with a low risk of spontaneous hemorrhage, particularly for tumors <1.5 cm. Systemic therapy has been associated with rare but serious intracranial bleeding events. Patient comorbidities (e.g., diabetes mellitus) and low preoperative KPS are significant risk factors for adverse perioperative outcomes. Targeted therapies represented by Belzutifan offer a new weapon for VHL patients. How to optimally integrate systemic drug therapy with local treatments and sequence them appropriately will be key to improving long term prognosis and quality of life. A hypothetical strategy—subtotal resection to reduce surgical morbidity followed by adjuvant Belzutifan to control residual tumor—could be considered in selected high-risk cases. However, this approach remains speculative, as no prospective data currently support neoadjuvant or adjuvant Belzutifan for CNS hemangioblastomas, and its use should be limited to investigational settings. We acknowledge that the rarity of VHL syndrome precludes large-scale prospective studies and robust evidence-based guidelines. Therefore, case reports and case series remain invaluable for accumulating clinical experience and sharing knowledge. Ultimately, all VHL patients should be enrolled in a standardized, multidisciplinary follow-up and management system to enable early diagnosis and treatment, thereby minimizing tumor-related mortality and disability.

## Data Availability

The original contributions presented in the study are included in the article/supplementary material, further inquiries can be directed to the corresponding author/s.
